# Detection of giant cytoplasmic inclusions in a pediatric patient with recurrent infections: a case report

**DOI:** 10.1515/almed-2023-0136

**Published:** 2024-02-05

**Authors:** Leire Saiz-Sierra, Anna Marull Arnall, Javier Nieto-Moragas, Meritxell Deulofeu, Orlando Jiménez Romero, Irene Mademont, María Obón Ferrer, María Teresa Serrando Querol

**Affiliations:** Laboratori Territorial ICS-IAS Girona, Hospital Universitari Doctor Josep Trueta, Girona, Spain; Comisión de Biología Hematológica de la SEQC, Girona, Spain; Department of Medical Sciences, Research Group of Clinical Anatomy, Embryology and Neuroscience (NEOMA), University of Girona, Girona, Catalonia, Spain; Laboratori Territorial ICS-IAS Girona, Hospital, Parc Hospitalari Martí i Julià, Salt, Girona, Catalunya, Spain

**Keywords:** giant inclusions, Chédiak-Higashi, genetic disorder

## Abstract

**Objectives:**

Giant inclusions in leukocytes is a common feature that can be observed in some infections but can be also related to rare genetic disorders such as Chédiak-Higashi syndrome (CHS). A differential diagnosis between these groups of diseases has to be performed using specific genetic tests. Clinical and pathological history is relevant for a diagnostic orientation due to the difficulty and specificity of the diagnostic confirmation.

**Case presenation:**

We present the case of a 3-years-old male patient with recurrent respiratory infections. It is important to highlight the presence of a lock of white hair on the front of the head and some hypopigmentation of the skin. In the blood smear, the presence of big cytoplasm granules in all the leukocytes, especially in neutrophils.

**Conclusions:**

CHS is an uncommon genetic disorder caused by the mutation in the *LYST* gene situated in chromosome 1q42.3 which codified for LYST protein. Molecular genetic testing also can be done to detect the biallelic variants in the *LYST* gene. It is essential to perform peripheral blood smears in the presence of changes in quantitative and/or qualitative values in the complete blood count as a first step in the diagnosis algorithm.

## Introduction

Chédiak-Higashi syndrome (CHS) is a rare autosomal recessive immune disease that was described by Beguzz (1943), Steinberk (1948), Chédiak (1952) and Higashi (1954) [1, 2]. CHS is caused by homozygous or heterozygous compound pathogenic genetic variants in the *LYST* gene (MIM *606897). The *LYST* gene is a lysosomal trafficking regulator gene [3, 4] located in the chromosomal locus 1q42.3, which encodes the protein LYST [2]. LYST is an adapter protein that has been involved in the regulation, fission and secretion of intracellular vesicles such as lysosome. It might regulate the traffic of the effectors related to exocytosis [4], [5], [6]. LYST has a role in the regulation of the size and number of the lytic granules of the cytotoxic T-cells and natural killer (NK) cells; moreover, it is involved in the pro-inflammatory cytokines production of the macrophages and dendritic cells by regulating the endosomal signaling pathways. It can also induce a down-regulation of protein Kinase, causing an immune dysfunction and abnormal phenotypes [4], [5], [6], [7].

Less than 500 cases of CHS have been reported in the literature [1]. The prevalence is unknown, probably due to the heterogeneity of the disorder and the lack of genetic confirmation of many of them. There are no documented differences between geographic regions or between continents. It is a syndrome that is generally diagnosed in the pediatric age because clinical appearance is usually observed in this period [8]. Pathogenic variants of the *LYST* gene are related to CHS (MIM #214500), characterized by partial oculocutaneous albinism, photophobia, nystagmus, coagulation disturbances, variable neurological manifestations and impaired immunity, especially of natural killer cells, predisposing to viral and bacterial infections. These signs are the result of functional abnormalities of the polynuclear cells, which contain large characteristic lysosomal inclusions [8], [9], [10]. The CHS has classic and atypical clinical form presentation. Patients with a classic form of the syndrome have more probability (up to 8.5 % of the patients) of developing an accelerate phase, also known as hemophagocytic lymphohistiocytosis (HLH), which can be fatal causing organ failure and death if not properly treated [4, 8, 11, 12]. Recurrent infections are related to the secretory granule protein missorting and impaired leukocyte exocytosis; on the other hand albinism is caused as a result of a tyrosinase deficiency in melanocytes due to exocytosis and not delivery to the melanosome [2].

CHS can be detected due to the presence of giant cytoplasm granules in cells like leukocytes in blood smear or bone marrow. Molecular genetic testing also can be done to detect biallelic pathogenic genetic variants in the *LYST* gene [13].

Recurrent infections are usually treated with empirical antibiotics but they might be treated according to the cultures. Other complications have to be treated depending on the patient’s evolution. The most appropriate treatment for the syndrome is hematopoietic stem cell transplantation or bone marrow transplantation; however, this does not treat the neurologic aspect of the disease [12].

We report a case of CHS in 3 years-old boy, whose first finding was the giant cytoplasm granules found in a routine blood test.

## Case presentation

The patient was a boy aged 3, who went to the primary care centre because of fever and symptoms of cold sores on the lips, mouth and tongue during the last 48 h. His relatives report that he had pain in eating. There was a prior history of recurrent respiratory infections that required antibiotic treatment. He had been followed since birth for his psychomotor abnormalities given his delay in psychomotor development.

During the physical examination multiple cold sores were observed in the oral cavity, palate and on the surface of the tongue. It was also observed the presence of a lock of white hair on the front of the head and some hypopigmentation of the skin.

As relevant information related to his medical history it had to be considered that their biological parents were consanguineous, increasing the possibility of a wide range of inherited alterations. Moreover, the pediatrician who monitored the patient points out that the older brother, a boy aged five at the time the clinical case was reported, presented a picture of intellectual disability and delayed psychomotor development (difficulty with fine motor skills, tremors and dimorphic features) that required periodic professional intervention. In the case of the older brother, there were previous results of molecular studies in which two chromosomal alterations were detected: a gain of 3 Mb of interstitial genetic material in the long arm of chromosome 5, which affects the chromosomal region 5q21.3, and a gain of interstitial genetic material of 2.9 Mb in the long arm of chromosome 9, which affects the chromosomal region 9q21.33q22.2. Both alterations were *de novo* and were classified as variants of uncertain significance, thus its clinical implication in the brother is currently unknown.

**Figure 1:**
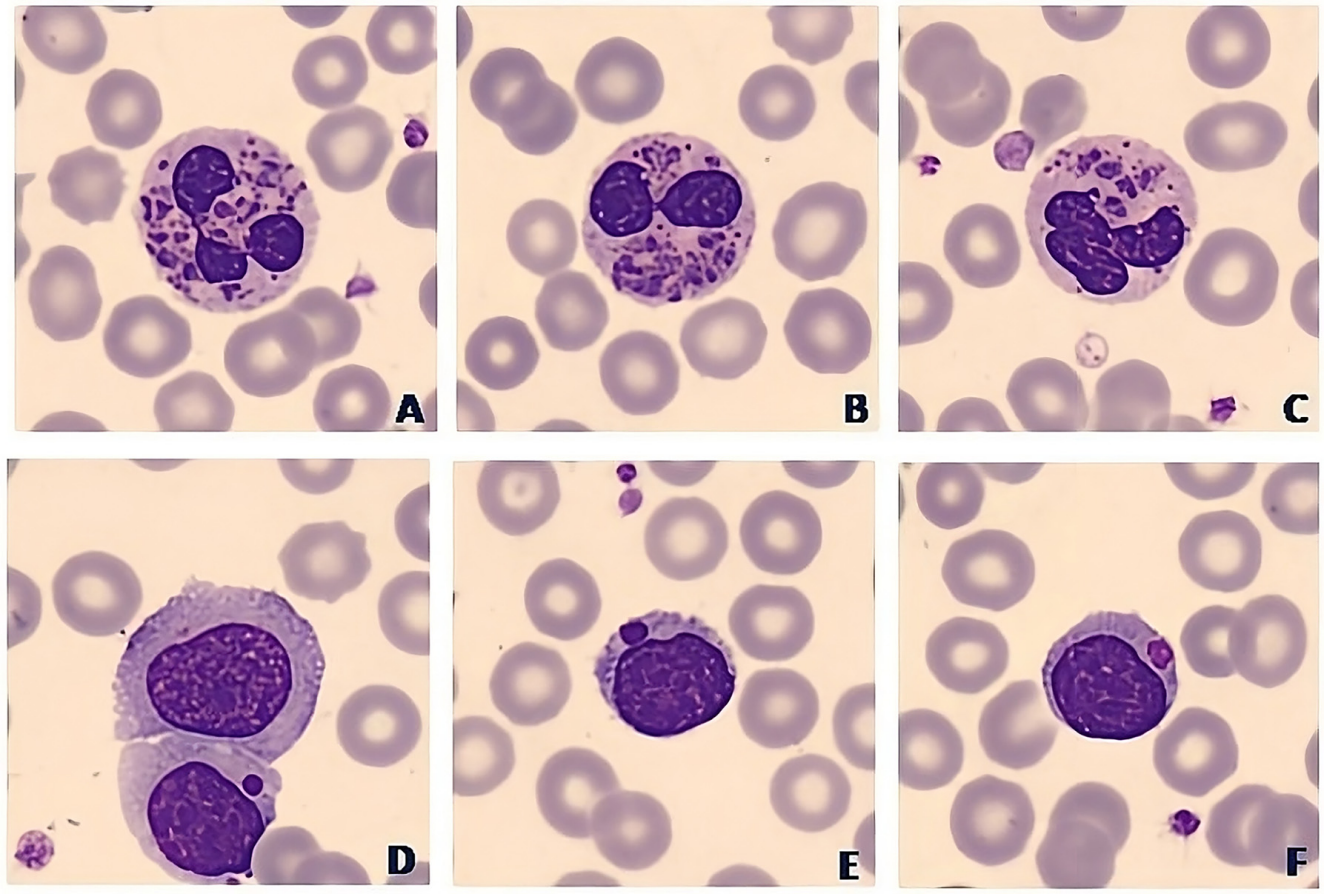
Cell images from peripheral blood smear of the patient in whom we can observe the giant inclusions in neutrophils (A–C), monocytes (D) and lymphocytes (E–F).

Laboratory results from the patient showed almost no alterations in the biochemical parameters measured. The C-reactive protein (CRP) was altered showing higher values than normal (CRP=7.1 mg/dL). Moreover, it was important to remark that thyroid-stimulating hormone (TSH) was not altered (TSH=5.03 mU/L). Hematological analysis revealed a moderate neutropenia (0.97 × 10^3^/µL) with monocytosis (1.72 × 10^3^/µL) that could be related to recurrent infections. As we can see in Figure 1, when looking under the microscope the blood smear, big cytoplasm granules/inclusions were present in all the leukocytes, especially in neutrophils, lymphocytes and eosinophils. These are findings that can be observed in many pathological conditions such as infections or in some inherited syndromes.

Given the suspicion of one of the inherited anomalies related to the presence of intracytoplasmic giant inclusions, we investigated the clinical history of the patient and the family. We looked at the patient’s own data that could be relevant in the diagnostic orientation and, therefore, in the selection of confirmation tests that would allow us to reach a diagnosis. Genetic and molecular results from the older brother leaded us to suspect that the clinical features in the patient might be explained by the presence of chromosomal alterations. The first diagnostic suspicion considering the cytological and clinical data of the patient was CHS. Thus, a custom phenotype-focused exome analysis was performed prioritizing changes detected in the genes associated with the observed hematological phenotype (*LYST*, *HPS1*, *MLPH*, *MYO5A*, and *RAB27A*) and genes related to the Human Phenotype Ontology (HPOs) terms: neutropenia (HP:0001875), language impairment (HP:0002463), delayed speech and language development (HP:0000750), poor fine motor coordination (HP:0007010), delayed fine motor development (HP:0010862), tremor (HP:0001337), and neurodevelopment delay (HP:0012758). The patient resulted homozygote for the likely pathogenic variant NM_000081.3:c.11173G>A p.(Gly3725Arg) in the *LYST* gene. Both parents and the brother presented the variant in heterozygosis, thus there was not clinical repercussion for any of them.

The patient remained well on prophylactic antibiotics till our last review of his clinical evolution; he was still waiting for an unrelated donor of stem cell transplantation.

## Discussion

Chédiak-Higashi syndrome (CHS) is a rare genetic disorder which inheritance pattern is autosomal recessive and is caused by a mutation in the *LYST* gene located on chromosome 1q42.3 and which codes for the LYST protein originally called LYST protein (lysosomal trafficking regulator gene) [11]. The probability for relatives varies depending on the relationship with the index case; the biological parents are usually heterozygous carriers of a mutation or pathogenic variants of the *LYST* gene. Two patients with CHS caused by a uniparental disomy of chromosome 1 have been described in the literature [14, 15]. For the offspring, there is a 25 % risk of not being affected by the mutation, a 25 % risk of having CHS, and a probability up to 50 % of being an asymptomatic heterozygous carrier. Heterozygous carriers are asymptomatic and are not at probability of developing the disorder. The direct descendants of the index case will be asymptomatic heterozygous carriers. Thus, genetic confirmation of the status of parents and siblings is necessary [2, 8].

The diagnosis of CHS must be initiated after the observation of giant granulation in the cytoplasm of leukocytes, especially granulocytes, both in peripheral blood and in bone marrow cells [2]. In these cases, genetic and molecular study is required to detect biallelic variants of the *LYST* gene. Next Generation Sequencing (NGS) is one of the most used strategies to detect intragenic small deletions and insertions and missense, nonsense and splice variants of the *LYST* gene [2, 3, 9]. In the case that we present here, a mutational analysis was carried out using Next-Generation sequencing and the changes detected in the genes associated with their clinical phenotype (*LYST, HPS1, MLPH, MYO5A,* and *RAB27A*) and the pathogenic changes detected that could be associated were analyzed and prioritized.

The presence of inclusions in the cytoplasm of leukocytes can be related to different pathological conditions such as bacterial infections (more often in neutrophils), viral infections (usually located in T-cells or NK cells) but also in some inherited immunodeficiency syndromes, CHS or Alder-Reilly syndrome in particular. Both inherited immunodeficiency’s can show abnormal giant inclusions so the differential diagnosis has to be considered. These cytological findings in the CHS have been ultrastructurally identified as giant lysosome-related organelles in the various cellular components (neutrophils, lymphocytes, eosinophils or monocytes) [6, 7]. The most relevant genetic conditions related to these microscopically findings are detailed in Table 1. Considering that there are many rare syndromes with genetic etiology and high clinical heterogeneity such as intellectual disability, neutropenia or immunodeficiency (recurrent infections) a differential diagnosis has to be done.

**Table 1:** Differential diagnosis in the appearance of giant cytoplasm granulation in peripheral blood cells.

Pathology	Characteristics	Cytology	Affected cells	Affected gene
Toxic granulation	Infection or inflammation, pregnancy, G-CSF			
Chédiak-Higashi	Immunodeficiency, Anemia, neutropenia, jaundice, neurological pathology, recurrent infections	Giant granules of variable staining	Leukocytes	*LYST*
Alder- Reily	Mucopolysaccharidosis	Coarse granulation similar to toxic	Neutrophils, eosinophils, lymphocytes and rarely monocytes	Myeloperoxidase gene (*MPO*)
Alterations related to MYH9	Thrombopenia and giant platelets	Similar to Döhle bodies	Neutrophils, eosinophils, monocytes, basophils	MYH9
Hermansky-Pudlak	Pulmonary fibrosis; albinism; hemorrhagic diathesis secondary to platelet dysfunction			*HPS1*
Griscelli	Hypomelanosis, hypopigmentation, grey hair, neurological and immunological alterations	Coarse eosinophilic granulation	Granulocytes, (neutrophils and eosinophils)	*RAB27A*

HPS1, Hermansky-Pudlak syndrome 1 protein; RAB27A, ras-related protein Rab-27A.

Thus, the predesigned panel performed in this case includes the changes in the genes of the similar syndromes such as Alder-Reily syndrome, *MYH9* genetic alterations, Mucopolysaccharidosis and neutropenia [12, 14].

As conclusions we might point out that it is essential to perform peripheral blood smears in the presence of changes in quantitative and/or qualitative values in the complete blood count; the observation of giant-sized granulation in leukocytes, especially in granulocytes, guides us towards the diagnosis of a group of diseases of genetic and hereditary etiology that it is necessary to know and diagnose in the index patient and his relatives and finally, diagnostic confirmation by cytogenetic and molecular biology is indicated when this pathological group is suspected in order to establish the confirmatory diagnosis and carry out family genetic counseling.
